# Efficacy of dialectical behavioral therapy DBT for couples with emotional dysregulation

**DOI:** 10.1192/j.eurpsy.2021.2073

**Published:** 2021-08-13

**Authors:** A. Rady, A. Abdelkareem, T. Mouloukheya, N. Elkholy

**Affiliations:** Psychiatry, Alexandria University School of Medicine, Alexandria, Egypt

**Keywords:** DERS scale, Dialectical behavioral therapy DBT, couple therapy, emotional dysregulation

## Abstract

**Introduction:**

Couple therapy continues to gain in stature as a vital component of mental health services. The linkage of relationship distress to disruption of individual emotional and physical well-being emphasizes the importance of improving and extending empirically based strategies for treating couple distress

**Objectives:**

To evaluate the efficacy of dialectical behavior therapy “DBT” in outpatients couples with emotional dysregulation

**Methods:**

Twenty couples presented with marital distress and at least one of them suffers from emotional dysregulation assigned at their convenience or according to immediate availability of treatment slot to a couple DBT group. Arabic version of DERS was used for assessment of emotional dysregulation before and after intervention. Dyadic Adjustment Scale was used for assessment of marital adjustment

**Results:**

Both male and female partners showed significant improvement in marital adjustment and emotional regulation. Female partner showed significant higher change amplitude in both scales. Female partners showed significant improvement in all DERS subscales except for (GOALS) subscale (significant decrease), while male partners showed significant improvement in (IMPULSE), (AWARNESS), (STRATEGIES) and (CLARITY) subscales

**Conclusions:**

Dialectical behavioral therapy for couples is an effective approach to couples with emotional dysregulation in one or both partners

**Disclosure:**

No significant relationships.

